# Functional Ophthalmic Factors Associated With Extreme Prematurity in Young Adults

**DOI:** 10.1001/jamanetworkopen.2021.45702

**Published:** 2022-01-28

**Authors:** Saurabh Jain, Peng Yong Sim, Joanne Beckmann, Yanyan Ni, Nabil Uddin, Bronia Unwin, Neil Marlow

**Affiliations:** 1 Ophthalmology Department, Royal Free London NHS Foundation Trust, London, United Kingdom; 2 Ophthalmology Department, Moorfields Eye Hospital NHS Foundation Trust, London, United Kingdom; 3 Academic Neonatology, University College London Elizabeth Garrett Anderson Institute for Women’s Health, University College London, London, United Kingdom; 4 Orthoptics Department, Cambridge University Hospitals NHS Foundation Trust, Cambridge, United Kingdom

## Abstract

**Importance:**

Children born preterm (<37 weeks’ gestation) have a higher risk of visual impairment and ocular morbidities compared peers born at full term. However, the long-term ocular sequelae in adulthood for those born extremely preterm (EP), who have the highest risk of neonatal retinopathy, are unknown.

**Objective:**

To evaluate visual function and ocular morbidity in young adults born EP compared with controls born full term.

**Design, Setting, and Participants:**

This prospective cohort study of a geographically based birth cohort in the UK and Ireland born from March 1 through December 31, 1995, included 128 participants aged 19 years (born at 22-25 weeks’ gestation) and 65 age-matched controls born at full term. Statistical analysis was performed from March 1, 2020, to November 26, 2021.

**Exposures:**

Participants underwent eye examinations as part of a comprehensive outcome evaluation.

**Main Outcomes and Measures:**

Best-corrected visual acuity, refractive status, contrast sensitivity, color vision, prevalence of strabismus and nystagmus, and patient-reported visual function, measured using the Health Utilities Index Mark 3.

**Results:**

The study comprised 128 participants (256 eyes; 68 female participants [53%]; mean [SD] age, 19.3 [0.5] years) and 65 age-matched controls born at full term (130 eyes; 40 female participants [62%]; mean [SD] age, 19.2 [0.5] years). Compared with control eyes, the mean (SD) best-corrected visual acuity among eyes in the EP group was significantly worse (monocular vision: −0.06 [0.14] logMAR in the control group vs 0.14 [0.38] logMAR in the EP group; *P* < .001; binocular vision: −0.14 [0.15] logMAR in the control group vs 0.06 [0.37] logMAR in the EP group; *P* < .001). Participants in the EP group had a significantly higher prevalence of strabismus (36% [46 of 127] vs 0%; *P* < .001), abnormal ocular motility (15% [19 of 125] vs 0%; *P* < .001), and nystagmus (13% [16 of 127] vs 0%; *P* < .001) than the control group. No significant differences between participants in the EP group and controls were observed for refractive error, contrast sensitivity, color vision, or patient-reported visual function. Among the participants in the EP group, 48% of eyes (120 of 250) had no retinopathy of prematurity (ROP), 39% (98 of 250) had ROP not requiring neonatal treatment, and 13% (32 of 250) received cryotherapy or laser ablation for ROP. Within the EP group, there was no significant difference in binocular visual function parameters, prevalence of ocular morbidity, and patient-reported visual function by neonatal ROP status.

**Conclusions and Relevance:**

Extreme prematurity is associated with an increased prevalence of visual and ocular deficits in young adulthood; this study suggests that, for individuals born EP, visual and ocular deficits appear to be partially independent of ROP status in the neonatal period but reports similar overall visual function.

## Key Points

**Question:**

What are the long-term ocular sequelae for young adults born extremely preterm (≤25 weeks’ gestation)?

**Findings:**

In this cohort study conducted in the UK and Ireland with 19 years of follow-up comparing 128 former extremely preterm infants with 65 age-matched full-term controls, extreme prematurity was associated with an increased prevalence of visual and ocular deficits. These deficits varied with, but were not fully explained by, neonatal retinopathy of prematurity status.

**Meaning:**

Extreme prematurity may have lifelong ocular implications that extend into adulthood; this study suggests that, for individuals born extremely preterm, ocular sequelae may be independent of the presence of neonatal retinopathy of prematurity and may be due to other causes.

## Teaser

This birth cohort study evaluates visual function and ocular morbidity in young adults born extremely preterm compared with controls born full term.

## Introduction

Children born preterm (<37 weeks’ gestational age) are at greater risk than their peers born at full term of developing visual impairment and ocular morbid conditions.^1,2,3,4^ The most common neonatal ophthalmic complication is retinopathy of prematurity (ROP), which is potentially vision threatening and remains a leading cause of childhood blindness worldwide, particularly among children born very preterm.^5^ However, individuals’ visual compromise from other ocular morbid conditions, such as refractive errors, strabismus, and cerebral visual impairment, has a significant effect on visual function and may negatively affect subsequent cognitive and psychological development.^4^

Many epidemiologic studies over the last few decades have concentrated on investigating the long-term ophthalmic outcomes among children with or without ROP.^3,6,7,8,9^ Although closely associated with prematurity, low birth weight and ROP do not fully account for consequent ophthalmic issues in preterm infants,^10^ and there remains a need to discern the association of preterm birth per se with long-term ophthalmic development.

To our knowledge, few studies have described the ophthalmic development of infants born extremely preterm (EP), who have the highest risk of ROP. Advances in neonatal care in the mid-1990s led to a shift of focus toward infants with extremely low gestational ages, who survived in greater numbers and had a high risk of neurosensory impairment.^11^ Contemporary data are therefore required to understand ophthalmic outcomes for infants born EP and their evolution into young adult life.

The purpose of this study was to evaluate visual function and ocular morbidity in a large well-characterized cohort of young adults who were born at 25 weeks’ gestation or less and to contrast their outcomes with those of full-term controls. We hypothesized that there would be an increased rate of visual and ocular abnormalities in EP births that persist into early adulthood, most frequent for those who had neonatal ROP.

## Methods

### Participants and Data Collection

The EPICure cohort comprises all infants born at 25 completed weeks or less of gestation in each of the 276 maternity units in the whole of the UK and Republic of Ireland from March 1 through December 31, 1995. To date, the EPICure cohort has been evaluated at ages 2.5 years,^12^ 6 years,^13^ and 11 years,^14^ with this study, EPICure@19, representing the latest follow-up at 19 years of age.^15^ Ethical approval was granted by the National Research Ethics Service South Central Committee–Hampshire, and all participants provided written informed consent (for those lacking capacity to consent, written assent was sought from a parent). This study adhered to the tenets of the Declaration of Helsinki.^16^ This report followed the Strengthening the Reporting of Observational Studies in Epidemiology (STROBE) reporting guideline for observational studies.^17^

EPICure study participants aged 18 to 20 years were invited for a comprehensive health assessment at the National Institute for Health Research University College London Hospital Clinical Research Facility, together with a comparison group born full-term who had been evaluated at 11 years of age. The EPICure cohort comprised individuals with or without neonatal ROP; among those with neonatal ROP, some experienced spontaneous resolution, and some received cryotherapy or laser ablation during the neonatal period. The comparison group comprised healthy volunteers born at full term, previously recruited at 6 and 11 years of age, who were classmates of the EPICure participants and, as a group, had no significant differences in key confounding factors, such as age, sex, and socioeconomic status.^11^

Of 306 known infants born EP at 19 years of age, 129 (42%; 68 female participants; mean [SD] age, 19.3 [0.5] years) participated in the 19-year follow-up, along with 65 of 153 control participants (43%; 40 female participants; mean [SD] age, 19.2 [0.5] years). Participants in the EP group and those in the control group who were not assessed at 19 years of age had either declined participation or did not respond to study invitations. EPICure@19 participants did not differ in baseline demographic characteristics from those lost to follow-up, as previously detailed (eTable in the Supplement).^18^

Ophthalmic evaluation was undertaken as part of a comprehensive clinical and psychological assessment between February 17, 2014, and October 31, 2015. This evaluation included assessment of best-corrected visual acuity (BCVA) using the crowded logMAR chart, color vision using the Ishihara 17-plate test, contrast sensitivity using the Pelli-Robson chart, refractive status using focimetry, and ocular motility using the cover test (reviewed by a single observer [J.B.]) and video-oculography (reviewed by 3 other observers [S.J., N.U., and B.U]). Strabismus was defined as the presence of a manifest deviation in the primary position at any distance, with or without glasses, confirmed by the cover test. Study participants also underwent anterior segment and dilated posterior segment examinations.

For this study, visual health status was evaluated using the Health Utilities Index Mark 3 (HUI-3).^19,20^ This is a well-validated, health-related, quality-of-life classification system and provides single-attribute scores of morbidity for 8 attributes: vision, hearing, speech, ambulation, dexterity, emotion, cognition, and pain.^19^ Participants scored the vision attribute, which has 6 levels of ability (level 1: can read ordinary newsprint and recognize a friend on the other side of the street, without glasses or contact lenses; level 2: can read ordinary newsprint and recognize a friend on the other side of the street, with glasses; level 3: can read ordinary newsprint with or without glasses but cannot recognize a friend on the other side of the street, even with glasses; level 4: can recognize a friend on the other side of the street with or without glasses but cannot read ordinary newsprint, even with glasses; level 5: cannot read ordinary newsprint or recognize a friend on the other side of the street, even with glasses; and level 6: cannot see at all).^20^

### Statistical Analysis

Statistical analysis was performed from March 1, 2020, to November 26, 2021. Refractive error was represented as spherical equivalent for statistical analysis. All data were investigated for normality to determine whether parametric or nonparametric methods should be used. The Mann-Whitney test was used to compare continuous data between eyes in the EP group and control eyes, and the comparisons shown are from mixed-effects models with a random participant effect to account for intereye correlation. The Kruskal-Wallis test was used to evaluate parameters across subgroups in the EP group. The χ^2^ test and the Fisher exact test were performed for categorical data. All statistical analyses were performed using IBM SPSS, version 23 (IBM Corp) and and Stata SE, version 14.1 (StataCorp LLC). All *P* values were from 2-sided tests and results were deemed statistically significant at *P* < .05.

## Results

A total of 386 eyes from 193 young adults were analyzed (256 eyes from 128 young adults in the EP group [between 22 and 25 completed weeks of gestation]; 68 female participants [53%]; mean [SD] age, 19.3 [0.5] years; and 130 eyes from 65 age-matched controls born at full term [≥37 weeks of gestation or greater], none of whom had any history of neonatal ROP; 40 female participants [62%]; mean [SD] age, 19.2 [0.5] years). In the EP group, 120 of 250 eyes (48%) had no neonatal ROP (EP-No-ROP), 98 of 250 eyes (39%) had ROP not deemed to require treatment in the neonatal period (EP-ROP-NT), and 32 of 250 eyes (13%) had ROP treated with neonatal cryotherapy or laser ablation (EP-ROP-T). There was no statistically significant difference in mean (SD) gestational age at birth within the 3 EP subgroups (EP-ROP-T group, 24.6 [0.8] weeks; EP-ROP-NT group, 25.0 [0.7] weeks; and EP-No-ROP group, 25.0 [0.8] weeks; *P* = .26).

Functional eye examinations were completed for 64 participants in the control group who attended the full evaluation; only 119 participants in the EP group (93%) had full assessments because some assessments were not possible (owing to the occurrence of nystagmus during monocular eye assessment or because the test procedures were unsuitable for people with learning disability; eFigure in the Supplement).

Compared with control eyes, the mean (SD) BCVA of eyes in the EP group was significantly worse (monocular BCVA: −0.06 [0.14] logMAR in the control group vs 0.14 [0.38] logMAR in the EP group; *P* < .001; binocular BCVA: −0.14 [0.15] logMAR in the control group vs 0.06 [0.37] logMAR in the EP group; *P* < .001), as was mean (SD) binocular contrast sensitivity (1.95 [0.00] in the control group vs 1.89 [0.20] in the EP group; *P* = .02) (Table 1). Monocular contrast sensitivity, color vision (scored out of a possible total of 17, the maximum number of color plates that can be read in the test), and spherical equivalent of refractive error were similar between eyes in the EP and control groups. Within the EP group, monocular BCVA was significantly worse in participants with neonatal ROP, whether it was treated or not (Figure). Mean (SD) monocular contrast sensitivity was significantly better (no treatment, 1.82 [0.22] log units vs treatment, 1.63 [0.07] log units; *P* < .001), whereas mean (SD) color vision was significantly worse (no treatment, 15.7 [1.80] vs treatment, 16.7 [0.60]; *P* = .01) in the group of participants who did not receive treatment for ROP compared with those who did receive treatment for ROP (Table 2).

**Table 1.** Visual Function Parameters at 19 Years of Age in Individual Eyes of Participants Born Extremely Preterm and Control Participants

Parameter	Extremely preterm group		Control group		*P* value^a^
	No./total No. (%)	Mean (SD)	No./total No. (%)	Mean (SD)	
BCVA, logMAR					
Monocular	248/256 (97)	0.14 (0.38)	128/130 (99)	−0.06 (0.14)	<.001^b^
Binocular^c^	124/128 (97)	0.06 (0.37)	64/65 (99)	−0.14 (0.15)	<.001^b^
Contrast sensitivity, log units					
Monocular	238/256 (93)	1.81 (0.26)	128/130 (99)	1.85 (0.16)	.19
Binocular^c^	119/128 (93)	1.89 (0.20)	64/65 (99)	1.95 (0.00)	.02^b^
Color vision (Ishihara plates)	246/256 (96)	15.9 (2.0)	128/130 (99)	15.7 (2.9)^d^	.51
Refractive error, D	92/126 (73)	−0.61 (3.67)	40/52 (77)	−1.33 (1.79)	.39

Abbreviations: BCVA, best-corrected visual acuity; D, diopter.

^a^ Derived from the Mann-Whitney test and mixed-effects models with a random participant effect to account for intereye correlation. These were subsequently adjusted with the Benjamini-Hochberg procedure to control the false discovery rate.

^b^ Statistically significant.

^c^ Both eyes from each participant were analyzed as a pair.

^d^ Scored out of a possible total of 17, the maximum number of color plates that can be read in the test.

**Figure.**
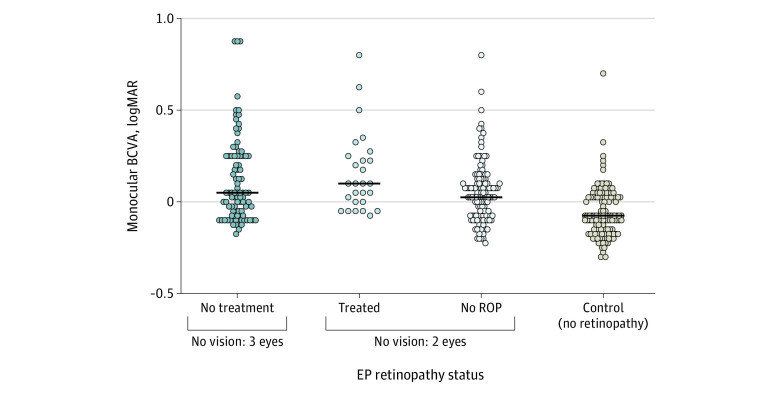
Monocular Best-Corrected Visual Acuity (BCVA) Among Participants Born Extremely Preterm (EP) by Neonatal Retinopathy of Prematurity (ROP) Status and Comparator Participants at 19 Years of Age Horizontal bars indicate the median for each group.

**Table 2.** Visual Function Parameters at 19 Years of Age of Participants Born EP by Neonatal ROP Status

Parameter	Participants born EP						*P* value^a^	
	EP-No-ROP		EP-ROP-NT		EP-ROP-T		EP-No-ROP vs EP-ROP	EP-ROP-NT vs EP-ROP-T
	No./total No. (%)	Mean (SD)	No./total No. (%)	Mean (SD)	No./total No. (%)	Mean (SD)		
BCVA, logMAR								
Monocular	118/120 (98)	0.08 (0.30)	94/98 (96)	0.15 (0.36)	30/32 (94)	0.34 (0.60)	.04^b^	.08
Binocular^c^	59/60 (98)	−0.06 (0.30)	47/49 (96)	0.12 (0.43)	15/16 (94)	0.11 (0.21)	.07	.24
Contrast sensitivity								
Monocular	118/120 (98)	1.85 (0.21)	84/98 (86)	1.82 (0.22)	30/32 (94)	1.63 (0.07)	.05	<.001^b^
Binocular^c^	59/60 (98)	1.91 (0.15)	42/49 (86)	1.87 (0.24)	15/16 (94)	1.85 (0.24)	.24	.69
Color vision (Ishihara plates)	116/120 (97)	15.9 (2.4)	94/98 (96)	15.7 (1.80)	30/32 (94)	16.7 (0.60)	.93	.01^b^
Refractive error, D	44/54 (81)	0.32 (4.06)	34/54 (63)	−1.74 (3.24)	12/18 (67)	−0.81 (2.79)	.08	.51

Abbreviations: BCVA, best-corrected visual acuity; D, diopter; EP, extremely preterm; EP-No-ROP, participants born EP without any signs of ROP; EP-ROP, participants born EP with ROP, treated or untreated; EP-ROP-NT, participants born EP with ROP that did not require neonatal treatment; EP-ROP-T, participants born EP with ROP treated with neonatal cryotherapy or laser ablation; ROP, retinopathy of prematurity.

^a^ Derived from the Kruskal-Wallis test and mixed-effects models with a random participant effect to account for intereye correlation. These were subsequently adjusted with the Benjamini-Hochberg procedure to control the false discovery rate.

^b^ Statistically significant.

^c^ Both eyes from each participant were analyzed as a pair.

Data on patient-reported visual function and the prevalence of ocular morbidity for young adults born EP and age-matched controls are summarized in Table 3. Most participants reported good functional vision (ie, level 1 or 2 on the HUI-3 vision attribute scale), with no significant differences between the EP group and the control group. Within the EP group, there was also no significant difference in visual function and prevalence of ocular morbidity by neonatal ROP status (Table 4). Four participants in the EP group, none of whom had received treatment for neonatal ROP, reported level 3 to level 5 vision on the HUI-3 vision attribute scale.

**Table 3.** Participant-Reported Visual Function and Other Ocular Morbidity at 19 Years of Age of Participants Born EP and Control Participants

Characteristic	No./total No. (%)		EP group vs control group	
	EP group	Control group	OR (95% CI)	*P* value^a^
HUI-3 vision attribute^b^				
Level 1	73/102 (72)	44/54 (81)	0.6 (0.2-1.4)	.33
Level 2	25/102 (25)	10/54 (19)	1.4 (0.6-3.7)	
Level 3	1/102 (1)	0/54	NA	
Level 4	2/102 (2)	0/54	NA	
Level 5	1/102 (1)	0/54	NA	
Level 6	0/102	0/54	NA	
Glasses or contact lens dependence	64/127 (50)	26/64 (41)	1.5 (0.8-2.9)	.20
Strabismus	46/127 (36)	0/64	NA	<.001^c^
Abnormal ocular motility	19/125 (15)	0/64	NA	<.001^c^
Nystagmus	16/127 (13)	0/64	NA	<.001^c^

Abbreviations: EP, extremely preterm; HUI-3, Health Utilities Index Mark 3; NA, not applicable; OR, odds ratio.

^a^ Derived from the χ^2^ test or the Fisher exact test.

^b^ Level 1: able to see well enough to read ordinary newsprint and recognize a friend on the other side of the street, without glasses or contact lenses; level 2: able to see well enough to read ordinary newsprint and recognize a friend on the other side of the street, but with glasses; level 3: able to read ordinary newsprint with or without glasses but unable to recognize a friend on the other side of the street, even with glasses; level 4: able to recognize a friend on the other side of the street with or without glasses but unable to read ordinary newsprint, even with glasses; level 5: unable to read ordinary newsprint and unable to recognize a friend on the other side of the street, even with glasses; and level 6: unable to see at all.

^c^ Statistically significant.

**Table 4.** Participant-Reported Visual Function and Other Ocular Morbidity at 19 Years of Age of Participants born EP by Neonatal ROP Status

Characteristic	No./total No. (%)			OR (95% CI)		*P* value^a^	
	EP-No-ROP	EP-ROP-NT	EP-ROP-T	EP-No-ROP vs EP-ROP	EP-ROP-NT vs EP-ROP-T	EP-No-ROP vs EP-ROP	EP-ROP-NT vs EP-ROP-T
HUI-3 vision attribute^b^							
Level 1	37/50 (74)	26/38 (68)	9/13 (69)	1.3 (0.5-3.4)	1.0 (0.2-4.4)	.52	.84
Level 2	11/50 (22)	10/38 (26)	4/13 (31)	0.8 (0.3-2.0)	0.8 (0.2-4.4)		
Level 3	1/50 (2)	0/38	0/13	NA	NA		
Level 4	1/50 (2)	1/38 (3)	0/13	1.0 (0.01-82.0)	NA		
Level 5	0/50	1/38 (3)	0/13	NA	NA		
Level 6	0/50	0/38	0/13	NA	NA		
Glasses or contact lens dependence	27/59 (46)	27/49 (55)	9/16 (56)	0.7 (0.3-1.5)	1.0 (0.3-3.4)	.28	.94
Strabismus							
Esotropia	12/58 (21)	12/49 (24)	4/16 (25)	0.8 (0.3-2.0)	1.0 (0.2-4.9)	.60	.97
Exotropia	7/58 (12)	5/49 (10)	3/16 (19)	1.0 (0.3-3.3)	0.5 (0.1-3.6)	.97	.37
Vertical	0/58	2/49 (4)	0/16	NA	NA	.50	.99
Not assessed	0/58	1/49 (2)	0/16	NA	NA		
Abnormal ocular motility	9/58 (16)	6/49 (12)	4/16 (25)	1.0 (0.3-3.0)	0.4 (0.1-2.4)	.98	.22
Nystagmus	8/59 (14)	6/49 (12)	2/16 (13)	1.0 (0.5-2.4)	0.7 (0.2-2.7)	.84	.98

Abbreviations: EP, extremely preterm; EP-No-ROP, participants born EP without any signs of ROP; EP-ROP, participants born EP with ROP, treated or untreated; EP-ROP-NT, participants born EP with ROP that did not require neonatal treatment; EP-ROP-T, participants born EP with ROP treated with neonatal cryotherapy or laser ablation; HUI-3, Health Utilities Index Mark 3; NA, not applicable; OR, odds ratio; ROP, retinopathy of prematurity.

^a^ Derived from χ^2^ test or Fischer exact test.

^b^ Level 1: able to see well enough to read ordinary newsprint and recognize a friend on the other side of the street, without glasses or contact lenses; level 2: able to see well enough to read ordinary newsprint and recognize a friend on the other side of the street, but with glasses; level 3: able to read ordinary newsprint with or without glasses but unable to recognize a friend on the other side of the street, even with glasses; level 4: able to recognize a friend on the other side of the street with or without glasses but unable to read ordinary newsprint, even with glasses; level 5: unable to read ordinary newsprint and unable to recognize a friend on the other side of the street, even with glasses; and level 6: unable to see at all.

The prevalence of strabismus in the EP group was significantly higher compared with the control group (36% [46 of 127] vs 0%; *P* < .001). Of these, 22% participants (28 of 127) had esotropia, 12% (15 of 127) had exotropia, and 2% (2 of 127) had a vertical deviation. There was also a significantly higher prevalence of abnormal ocular motility (15% [19 of 125] vs 0%; *P* < .001) and nystagmus (13% [16 of 127 vs 0%; *P* < .001) in the EP group compared with the control group (Table 3). Within the EP group, no significant difference was detected for the prevalence of ocular morbidity when grouped by neonatal ROP status (Table 4).

## Discussion

To our knowledge, this study is the first to evaluate visual function and ocular morbidity in young adults born EP. Our findings demonstrate overall worse monocular and binocular BCVA and binocular contrast sensitivity in participants in the EP group compared with control participants who were born full term. This finding is unsurprising given the already strong body of evidence showing a higher prevalence of reduced visual acuity (VA) in children and adolescents born preterm with or without low birth weight.^3,6,21,22,23,24^ Our results support the notion that previous suggestions of a delay in visual development are incorrect and that visual problems persist into later adult life. Despite this finding, most participants in the EP group reported good visual function.

Compared with the study by O’Connor and colleagues,^6^ which investigated visual function in a less premature cohort (with mean gestational age of 31 weeks) at 11 years of age, we observed worse monocular and binocular BCVA among participants in the EP group than among the cohort of O’Connor et al^6^ (monocular BCVA: –0.02 logMAR in the less premature cohort of O’Connor et al^6^ vs 0.14 logMAR in the EP group; binocular BCVA: –0.12 logMAR in the less premature cohort of O’Connor et al^6^ vs 0.06 logMAR in the EP group), suggesting an association between increasing prematurity and VA deficit. Direct comparison with the findings of O’Connor et al^6^ may, however, be problematic given the difference in maturity and the earlier recruitment period before the introduction of key treatments for ROP and of important respiratory interventions, such as antenatal corticosteroid and surfactant replacement therapy, all of which may have improved outcomes.

Compared with previous studies reporting a positive correlation between the severity of neonatal ROP and degree of VA deficit,^6,25,26,27^ we observed a significant difference in monocular but not binocular BCVA when participants were grouped by ROP status and in monocular but not binocular contrast sensitivity in the EP-ROP-NT group compared with the EP-ROP-T group. Some of the disparity may be explained by the inclusion of intereye correlation in our analyses, which reduces the risk of falsely significant results.^28^ In addition, our data from individual eyes within the EP group show a large range of VA scores, suggesting that the previously reported positive correlation between ROP severity and VA deficit is not necessarily associated with a similar correlation in the eyes in the EP group.

Although the presence of neonatal ROP might account for the VA deficit in individuals born EP, the cause of reduced VA in eyes without ROP among those born EP remains unclear. These ROP-independent factors are likely to be complex and multifactorial. Several hypotheses have been proposed, including cone damage secondary to prolonged exposure to phototherapy for neonatal jaundice^29^ and alterations to the retina and optic nerve from prenatal endotoxin exposure through intrauterine infections associated with preterm delivery.^30^ The latter theory of disruption in retinal development is corroborated by recent optical coherence tomography findings from the EPICure@19 cohort demonstrating a correlation between increased ganglion cell layer thickness and worse BCVA, which suggests delayed foveal maturation.^31^ Reduced VA may also be associated with neonatal neurologic insults, including ischemic brain lesions and intraventricular hemorrhage, in children born preterm.^32,33,34^

Despite the higher rate of visual impairment associated with extreme prematurity, we found no difference in perceived visual function among young adults born EP compared with that of controls born at full term; most participants reported good functional vision. This discrepancy between objective measurement of vision and perceived visual function indicates that the former is not necessarily associated with overall visual effect in a real-world setting. There is evidence that emotional and psychological factors may be associated with the level of perceived visual ability.^35^ Our finding may also reflect a compensatory mechanism for visual deficit among individuals born EP through multisensory adaptation and integration in early life, which is associated with a more positive outcome for perceived visual function.

There is no disputing the increased prevalence of strabismus among children born preterm, with reported rates varying between 10% and 22%.^36,37,38^ Our results not only further attest to this observation but also show a higher rate (36%) of strabismus in the EP group, suggesting a positive association between the degree of prematurity and the prevalence of strabismus. By contrast, to our knowledge, there is a paucity of data with regard to strabismus type in infants born preterm. The only study, to our knowledge, to investigate strabismus type in infants born preterm reported a 1:1 ratio of esotropia to exotropia in a cohort with low birth weight (with a mean gestational age of 31 weeks).^39^ In comparison, we found a near 2:1 ratio of esotropia to exotropia, which may suggest a different cause of strabismus among infants born EP. We also show a higher prevalence of abnormal ocular motility (15%) and nystagmus (13%) in our cohort compared with that reported for infants born less premature,^40^ further reaffirming the higher burden of ocular morbidity in those born EP.

Previous studies have found that the higher prevalence of visual deficits and ocular morbid conditions among infants born preterm are either wholly or in part associated with ROP.^6,41^ Although this finding would make sense intuitively, our results indicate that the same may not be necessarily true in those born EP. Given the mixed subgroup differences among the EP group, it remains unclear as to the extent that ROP is responsible for the described visual morbidity. Alternatively, there may be a significant association from other recognized sequelae of prematurity, such as neurologic damage, which would warrant further investigation. It is important to evaluate this in more recent cohorts with more detailed data on ROP status and perinatal events, which were not available in this epidemiologic cohort.

### Limitations

This study has some limitations. One of the key problems with longitudinal studies is participant attrition. Observing a group of individuals over 20 years is challenging, and the reported results include data from less than half of the participants in both the EP group and the control group. Many participants had just commenced in higher education and thus may have been reluctant to travel to London for 2 days of evaluation.

Nonetheless, comparing the sample with those who did not participate, the 2 groups did not differ on a range of neonatal measures. At 2.5 years of age, 7 children in the EP group were blind or saw light only and were considered to have severe visual impairment.^12^ Only 1 of these children (with comorbid severe learning difficulties) was evaluated at 19 years of age. As can be seen from the dropout analysis (eTable in the Supplement), participants in the EP group seen at 19 years of age had lower rates of cognitive impairment and overall disability at 11 years of age. Hence, given the association of visual outcomes with neurodevelopmental problems, we may have somewhat underestimated the overall visual impairment in this group.

## Conclusions

This study demonstrates the increased prevalence of visual and ocular deficits in a population of individuals born EP and suggests that children born EP may remain “preterm for life,” with ophthalmic sequelae persisting into adulthood. Comparison, both directly with full-term controls and indirectly with less premature cohorts, indicates that individuals born EP have the greatest risk for visual and ocular morbidity, although their perceived visual health status remains good overall. Although ROP remains a risk factor, the mixed findings in our study emphasize that all individuals born EP are at risk of visual and ocular morbidity irrespective of ROP status. These findings provide important insight into the ophthalmic outcome of EP birth on a population level and have implications for the screening and management of such deficits as they evolve over a lifespan.

## Notes

Supplement. **eTable.** Characteristics of Extremely Preterm Participants and Controls Attending for Evaluation at 19 Years of Age **eFigure.** Flow Chart of Extremely Preterm and Comparator Participants Showing the Completeness of the Individual Ophthalmic Assessments at 19 Years of Age
