# Error in Byline

**DOI:** 10.1001/jamanetworkopen.2022.11768

**Published:** 2022-04-21

In the Original Investigation titled “Functional Ophthalmic Factors Associated With Extreme Prematurity in Young Adults,”^1^ published January 28, 2022, an academic degree was missing after the third author’s name in the byline. It should have appeared as Joanne Beckmann, MBBS, MSc. This article has been corrected.^1^
