# Supplementary material for: Functional Ophthalmic Factors Associated With Extreme Prematurity in Young Adults
Source: JAMA Netw Open. 2022 Jan 28;5(1):e2145702. doi: 10.1001/jamanetworkopen.2021.45702 (PMC8800073; doi:10.1001/jamanetworkopen.2021.45702)
Supplement: Supplement. — eTable. Characteristics of Extremely Preterm Participants and Controls Attending for Evaluation at 19 Years of Age eFigure. Flow Chart of Extremely Preterm and Comparator Participants Showing the Completeness of the Individual Ophthalmic Assessments at 19 Years of Age [file jamanetwopen-e2145702-s001.pdf]

Supplementary Online Content

Jain S, Sim PY, Beckmann J, et al. Functional ophthalmic factors associated with extreme prematurity in young adults. *JAMA Netw Open.* 2022;5(1):e2145702. doi:10.1001/jamanetworkopen.2021.45702

eTable. Characteristics of Extremely Preterm Participants and Controls Attending for Evaluation at 19 Years of Age
eFigure. Flow Chart of Extremely Preterm and Comparator Participants Showing the Completeness of the Individual Ophthalmic Assessments at 19 Years of Age

This supplementary material has been provided by the authors to give readers additional information about their work.

© 2022 Jain S et al. *JAMA Network Open.*

eTable. Characteristics of Extremely Preterm Participants and Controls Attending for Evaluation at 19 Years of Age

**Variable**		**EP assessed n=129**	**EP not assessed n=177**	**Controls assessed n=65**	**Controls not assessed n=88**
**Male Sex**	n/N (%)	61/129 (47)	87/177 (49)	25/65 (39)	39/88 (44)
**Birth characteristics (n=306)**					
**Birth weight, grams**	Mean (SD)	741 (122) [n=129]	751 (109) [n=177]		
**Birthweight z-score**	Mean (SD)	-0.2 (0.8) [n=126]	-0.2 (0.8) [n=177]		
**Gestational age, weeks**	Mean (SD)	24.9 (0.8) [n=129]	25.0 (0.6) [n=177]		
22 weeks n/N (%)		2/129 (2)	0/177 (-)		
23 weeks n/N (%)		13/129 (10)	13/177 (7)		
24 weeks n/N (%)		37/129 (29)	60/177 (34)		
25 weeks n/N (%)		77/129 (60)	104/177 (59)		
**Any retinopathy**	n/N (%)	65/129 (50)	111/177 (63)		
ROP no treatment		49	82		
ROP treated with laser/cryotherapy		16	29		
**Cranial Ultrasound Abnormal**	n/N (%)	89 (69)	123 (69)		
Severe abnormality [b]		22 (17)	49 (28)		
**Bronchopulmonary dysplasia** [c]	n/N (%)	98/129 (76)	140/186 (74)		
**Evaluated at 2.5 years (n=280)**					
**BSID-II MDI** [d]	Mean (SD)	84 (13) [n=117]	80 (15) [n=130]		
**BSID-II MDI <70**	n/N (%)	15/117 (13)	27/130 (20.8)		
**Neurodevelopmental impairment** [e.]	n/N (%)	57/126 (45)	78/154 (50.6)		
**Evaluated at 11 years (EP: n=218; Controls: n=153)**					
**KABC MPC** [f]	Mean (SD)	86 (16) [n=121]	81 (19) [n=95]	106 (11) [n=65]	103 (11) [n=88]
**Cognitive impairment** [g]	n/N (%)	42/121 (35)	44/97 (45)	0/65 (-)	2/88 (2)
**Neurodevelopmental impairment** [h]	n/N (%)	50/121 (41)	47/97 (49)	0/65 (-)	2/88 (2)
**Maternal education at 11 years** [i]					

© 2022 Jain S et al. *JAMA Network Open*.

Low n/N (%)	7/102 (7)	14/92 (15)	2/50 (4)	5/49 (10)
Intermediate n/N (%)	88/102 (86)	71/92 (77)	38/50 (76)	41/49 (84)
High n/N (%)	7/102 (7)	7/92 (8)	10/50 (20)	3/49 (6)

[a]Denominators: N=306 EP survivors at 19 years; N=153 controls assessed at 11 years.

[b]Severe scan abnormalities: cystic parenchymal changes or ventriculomegaly.

[c]Receipt of supplemental oxygen at 36 weeks post menstrual age.

[d]BSID-II MDI, the Bayley Scales of Infant Development, 2nd edition, Mental Development Index.

[e]Neurodevelopmental impairment classified as one or more of cognitive, vision, motor or hearing impairment; cognitive impairment BSID-II MDI <70.

[f] KABC MPC, the Kaufman-Assessment Battery for Children 1st Edition, Mental Processing Composite.

[g]Cognitive impairment classified as a KABC MPC score more than 2 SD below the mean score of the term-born controls.

[h]Neurodevelopmental impairment classified as one or more of cognitive KABC MPC <-2SD of controls, vision, motor or hearing impairment.

[i]Maternal education collected by parent report and classified using the International Standard Classification of Education (ISCED): (1) low level: equivalent to ISCED 0 to 2; (2) Mediumlevel: equivalent to ISCED 3 to 5; (3) High level: equivalent to ISCED 6 to 10.

© 2022 Jain S et al. *JAMA Network Open*.

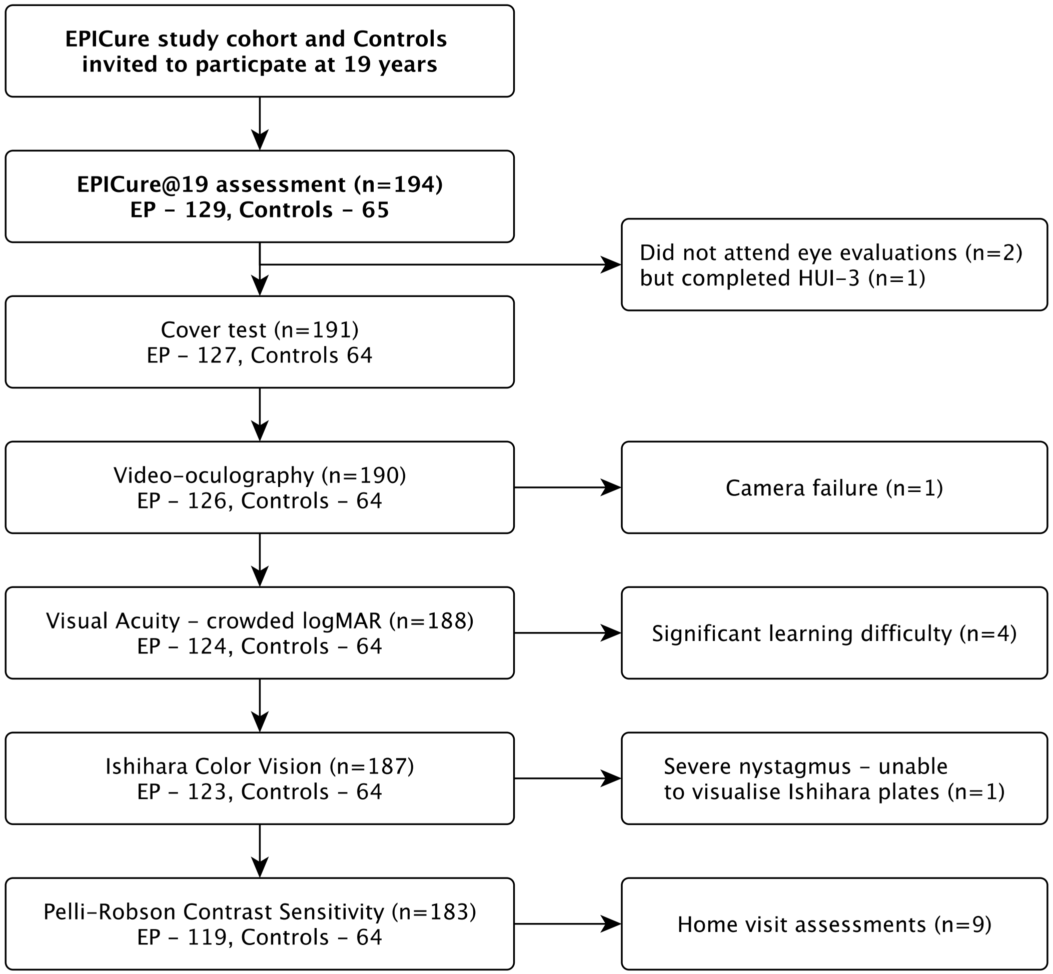

eFigure. Flow Chart of Extremely Preterm and Comparator Participants Showing the Completeness of the Individual Ophthalmic Assessments at 19 Years of Age

© 2022 Jain S et al. *JAMA Network Open.*
